# Supplementary material for: 4-Methylumbelliferone Inhibits Cancer Stem Cell Activation and Overcomes Chemoresistance in Ovarian Cancer
Source: Cancers (Basel). 2019 Aug 15;11(8):1187. doi: 10.3390/cancers11081187 (PMC6721459; doi:10.3390/cancers11081187)
Supplement: Supplementary file 1 [file cancers-11-01187-s001.pdf]

cancers

Supplementary Materials: The following are available online Figure S1-S3, Table S1-S4

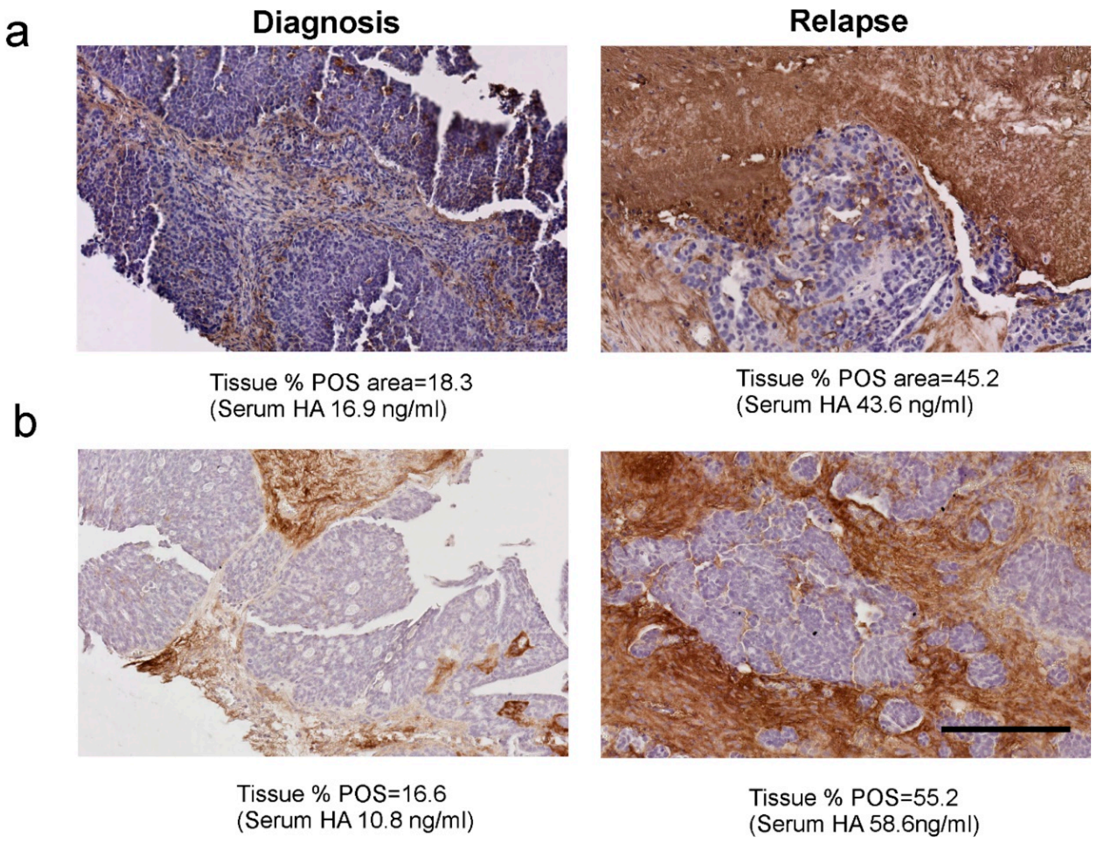

Figure S1. HA staining in matched tissues from two patients (A & B) at diagnosis and at relapse with chemoresistant disease. HA quantitation (% POS area, arbitrary units) and corresponding serum HA levels and are shown below the images. Scale bar= 100µm. All images are the same magnification

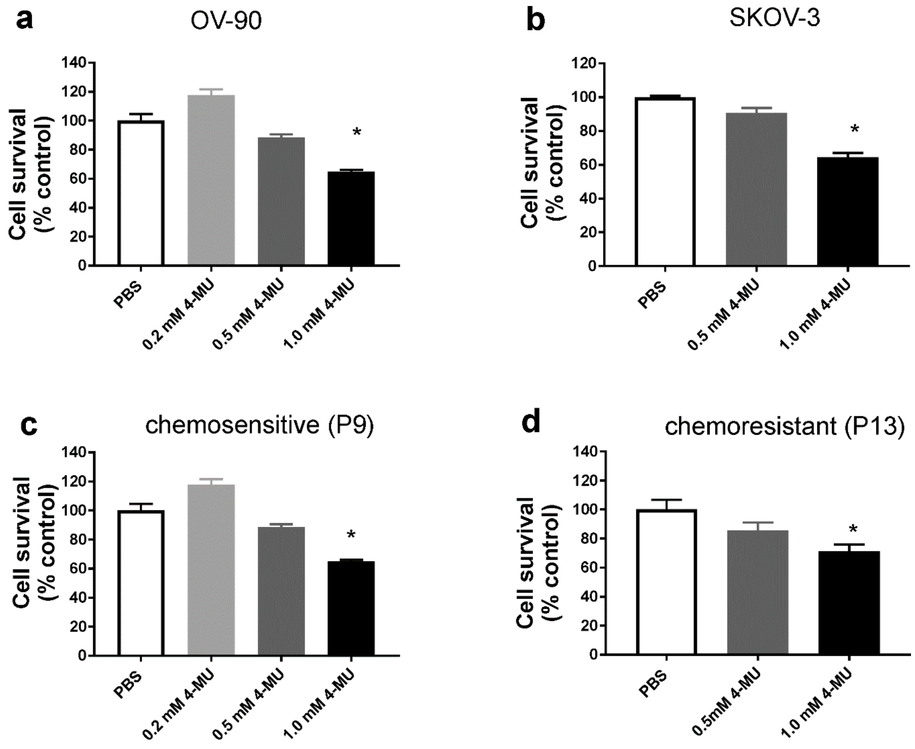

Figure S2. Effect of increasing concentrations of 4-MU on ovarian cancer cell survival. a). OV-90, **b**) SKOV-3, **c**) chemosensitive primary cells (P9) and **d**) chemoresistant primary cells (P13). Cells were treated with 4-MU (0.1-1mM) for 72hr. Data is expressed as % of PBS control from 1-2 independent experiments performed in quadruplicate. *, significantly different from control ($P < 0.05$, One Way ANOVA, Tukey's multiple comparisons test)

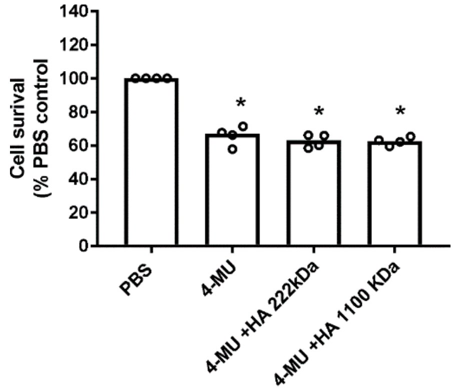

Figure S3. Exogenous HA does not reverse effects of 4-MU on ovarian cancer cell survival. Primary ovarian cancer cells were treated with 4-MU (1mM) for 72hr ± exogenous HA (10µg/ml, 222 kDa (Contripro C CO) and 1100 kDa (Caref laboratories). Data is from primary ovarian cancer cells (n=4) expressed as % of PBS control from 1 independent experiment performed in quadruplicate. *, significantly different from control ($P < 0.05$, One Way ANOVA, Tukey's multiple comparisons test).

Table S1. Summary of clinical and pathological characteristics of patient serum cohorts.

Patient	Age at Diagnosis	Diagnosis	Stage	Grade	Progression-free survival (months)	Overall survival (months)	Chemosensitive relapse	Patient status at last follow-up
1	65	Serous ovarian carcinoma	2B	-	16.8	96.5	Yes	Alive[a]
2*	43	Peritoneal carcinoma	3C	3	28.6	61.2	Yes/No	Alive
3*	82	Peritoneal carcinoma	3C	-	8.8	58.6	Yes/No	Cancer[b]
4	77	Serous papillary carcinoma of the ovary	3C	3	10.8	67.50	Yes	Alive
5	78	Serous papillary carcinoma of the fallopian tube	3C	3	11.7	63.60	Yes	Alive
6	46	Papillary serous carcinoma of the ovary	3C	3	11.4	26.4	Yes	Alive
7	60	Serous papillary carcinoma of the ovary	3C	3	16.5	47.50	Yes	Alive
8	54	Serous peritoneal carcinoma	3C	3	9.2	29.0	No	Cancer
9	65	Serous ovarian carcinoma	1C	3	46.10	70.80	No	Cancer
10	46	Serous papillary carcinoma ovary	3C	3	51.0	53.0	No	Alive
11	81	Serous papillary carcinoma peritoneum	4	-	13.5	46.6	No	Cancer
12	69	Papillary serous carcinoma of the ovary	3A	3	11.6	28.2	No	Cancer
13	78	Serous papillary carcinoma of the ovary	3A	3	9.6	18.7	No	Cancer
14	66	Serous carcinoma of ovary/ peritoneum	3C	3	12.8	21.1	No	Cancer

* 1st relapse chemosensitive and subsequent chemoresistant relapse

[a]Alive=alive at last follow-up, [b]Cancer= death due to ovarian cancer

Table S2. Summary of clinical and pathological characteristics of the primary ovarian cancer cells established from patient ascites.

Patient	Age at Diagnosis (years)	Stage at Diagnosis	Diagnosis	Chemosensitive
1	46	IIIC	Serous papillary carcinoma of the ovary	Yes
2	66	IIIC	Serous carcinoma of ovary/peritoneum	Yes
3	72	IIIC	Serous papillary carcinoma of the ovary	Yes
4	46	IIIC	Serous papillary carcinoma of the ovary	Yes
5	61	IIIA	Papillary serous carcinoma of the ovary	Yes
6	58	IIIC	Serous papillary carcinoma of the ovary	Yes
7	80	IIIC	Serous papillary carcinoma of the ovary	Yes
8	60	IIIC	Serous papillary carcinoma of the ovary	No
9	80	IIIC	Peritoneal carcinoma	No
10	47	IIIC	Recurrent serous carcinoma of the ovary	No
*11	59	1A	Recurrent serous tubal	No
12	47	IIIC	Recurrent serous peritoneal cancer	No
13	81	IV	Recurrent serous peritoneal carcinoma	No
14	43	IIC	Recurrent serous peritoneal carcinoma	No
*15	59	IA	Recurrent serous tubal	No
**16	48	IV	Recurrent serous peritoneal	No
17	69	IIIA	Recurrent serous ovarian cancer	No
**18	48	IV	Recurrent serous peritonea	No
19	57	-	Recurrent serous carcinoma	No

* Ovarian cancer cells were derived from the same patient following an interval of 21 months

** Ovarian cancer cells were derived from the same patient following an interval of 1 month

Table S3. Summary of Taqman gene probes used for qRT-PCR.

Gene	Catalogue number
HAS1	Hs00987417_g1
HAS2	Hs00193435_ml
HAS3	Hs00193436_ml
HYAL1	Hs00201046_m1
HYAL2	Hs01117343_g1
ALDH1A1	Hs00946916_m1
PROM1	Hs00195682_m1
CD44	Hs01075864_m1
ABCG2	Hs01053790_m1
ACTB	4333762F

Table S4. Summary of clinical and pathological characteristics of ovarian cancer tissue explant tissue cohort.

Patient	Age at Diagnosis (years)	Stage at Diagnosis	Grade	Diagnosis	Chemosensitive
1	66	IIIC	3	Serous papillary carcinoma of the ovary	No chemotherapy
2	51	IIIC	3	Serous papillary carcinoma of the peritoneum	No
3	66	IIIC	3	Serous papillary carcinoma of the ovary	Yes
4	80	IIIC	3	Primary peritoneal carcinoma	Yes
5	55	IIIC	3	Serous carcinoma of peritoneum	No
